# The Incidence of West Nile Disease in Russia in Relation to Climatic and Environmental Factors

**DOI:** 10.3390/ijerph110201211

**Published:** 2014-01-23

**Authors:** Alexander E. Platonov, Vladimir A. Tolpin, Kristina A. Gridneva, Anton V. Titkov, Olga V. Platonova, Nadezhda M. Kolyasnikova, Luca Busani, Giovanni Rezza

**Affiliations:** 1 Central Research Institute of Epidemiology, Novogireevskaya Street 3A, Moscow 111123, Russia; E-Mails: gridneva@pcr.ru (K.A.G.); anton.titkov@bk.ru (A.V.T.); oplatonova@pcr.ru (O.V.P.); kolyasnikova@pcr.ru (N.M.K.); 2 Space Research Institute, Profsoyuznaya Street 84/32, Moscow 117997, Russia; E-Mail: tolpin@d902.iki.rssi.ru; 3 Istituto Superiore di Sanità, Viale Regina Elena 299, Rome 00161, Italy; E-Mails: luca.busani@iss.it (L.B.); giovanni.rezza@iss.it (G.R.)

**Keywords:** West Nile fever, Russia, climate, ecology, prognosis, decision trees

## Abstract

Since 1999, human cases of West Nile fever/neuroinvasive disease (WND) have been reported annually in Russia. The highest incidence has been recorded in three provinces of southern European Russia (Volgograd, Astrakhan and Rostov Provinces), yet in 2010–2012 the distribution of human cases expanded northwards considerably. From year to year, the number of WND cases varied widely, with major WND outbreaks in 1999, 2007, 2010, and 2012. The present study was aimed at identifying the most important climatic and environmental factors potentially affecting WND incidence in the three above-mentioned provinces and at building simple prognostic models, using those factors, by the decision trees method. The effects of 96 variables, including mean monthly temperature, relative humidity, precipitation, Normalized Difference Vegetation Index, *etc.* were taken into account. The findings of this analysis show that an increase of human WND incidence, compared to the previous year, was mostly driven by higher temperatures in May and/or in June, as well as (to a lesser extent) by high August-September temperatures. Declining incidence was associated with cold winters (December and/or January, depending on the region and type of model). WND incidence also tended to decrease during year following major WND outbreaks. Combining this information, the future trend of WND may be, to some extent, predicted, in accordance with the climatic conditions observed before the summer peak of WND incidence.

## 1. Introduction

West Nile fever/neuroinvasive disease (WND) is caused by infection with West Nile virus (WNV), which belongs to the family *Flaviviridae*, genus *Flavivirus.* Clinical manifestations of WNV infection in humans are very diverse. Seroprevalence studies suggest that, in recent WND epidemics in the US, ~80% of infections were asymptomatic; ~20% presented as a dengue-like or flu-like viral syndrome, which is generally self-limited; and <1% led to neuroinvasive disease [1]. Elderly people and those on immunosuppressive drugs are at highest risk. During recent European and American outbreaks, serosurveys found nearly identical WNV infection rates in every age-group, whereas rates of neuroinvasive disease and case-fatality rate increased substantially with age; that is, the risk of developing severe WND is related to host susceptibility rather than exposure.

WNV is enzootic throughout Africa, Australia, west and central Asia, the Middle East, and the Mediterranean area, with occasional foci in Northern Europe and Siberia up to 55°N [2,3,4,5,6,7]. Following its introduction into the Western hemisphere in 1999, WNV rapidly disseminated across the USA and southern Canada and more recently has been reported in Mexico, the Caribbean and South America [4,8]. 

Phylogenetic analysis showed the existence of at least seven distinct lineages of WNV strains differing in geographical distribution and, possibly, in virulence and ecology. Isolates from the Americas, North, West and Central Africa, the Middle East, Eurasia, and Australia have been grouped in lineage 1, whereas isolates from South, West, and Central Africa and Madagascar constitute lineage 2 [9]. Since 2004 lineage 2 WNV strains were found also in Eurasia, first in Hungary in a goshawk (2004) and after in Southern Russia in humans (Rostov City in 2004 and Volgograd City in 2007) [10,11,12]. From 2010 onward most of the human WND cases observed in Europe (in Greece, Balkans, Italy, Russia, *etc.*) were caused by lineage 2 WNV strains [12,13,14,15,16].

In nature WNV is transmitted among birds by culicine mosquitoes. Recent studies in the United States have found infection in >1,300 species of birds and >60 species of mosquitoes [8,17]. The waterfowl, especially cormorants and storks, are the elements of an enzootic “sylvatic” cycle including ornithophilic mosquitoes, particularly *Cx. pipiens*, as a vector. Members of the order Passeriformes (jays, blackbirds, finches, warblers, sparrows, crows) seem to be also important in maintaining the virus in nature because of their high viraemias. Moreover, many of these species are synanthropic, that is ecologically associated with humans in urban and peri-urban areas contributing to an “urban cycle” of the infection [17,18,19].

Because of their presumed low and transient viraemias, humans and horses are not considered important in the natural transmission cycle. Different mosquito species may act as “bridging vectors” transmitting the virus to humans [17,18]. Humans may be infected both in rural and urban environments, but during recent WND outbreaks in Europe most patients lived in large cities [3,13,14,15,20]. The association of WND with flooded apartment building basements stressed the importance of indoor populations of autogenous *Cx. pipiens* form *molestus* mosquitoes [21,22,23].

The incidence of WND is seasonal in the temperate zones of Eurasia, the Mediterranean Basin, and North America, peaking from July through October [17,20]. In temperate climates the WND incidence is related to the weather, although it is uncertain how temperature and rainfall influence epidemic transmission [24]. In 1999, there was a large outbreak of WND in Southern Russia (>500 cases in the Volgograd Province). In 2000–2004, the WND incidence decreased steadily to zero, but a new outbreak occurred in 2007. The analysis of historical climate data for Volgograd from 1900 to present has shown that the years 1999 and 2007 were the hottest ones due to a very mild “winter” (December–March) and a hot “summer” (June–September) [2,3]. Similar conclusions were reached by analyzing weather conditions during the WNV outbreak in Israel in 2000 [25]. It is clear, however, that there is a complex interplay of viral, avian, mosquito, human, and climatic factors that contribute to epidemic/epizootic transmission of WNV [24,26]. For example, avian herd immunity developed during a year of high WNV activity might result in decrease in transmission during the following season.

Either the number of human WND cases or the prevalence and WNV infection rate of mosquitoes were used as a measure of the WND risk in certain conditions [25,27,28,29,30,31,32,33]. An increase in temperature in the 18–30 °C range shortens the gonotrophic period (GP) of *Culex pipiens* increasing the frequency of mosquito-host contact and therefore infection/transmission. Also the reproduction of WNV in infected mosquitoes increases along with temperature rise [34,35,36]. For example, so-called extrinsic incubation period (EIP) of WNV NY99 strain in *Cx. tarsalis* mosquitoes decreased from 30 days at 18 °C to 10 days at 26 °C [36]. Supposedly, this results in intensifying of spreading the WNV infection [2,3]. The transmission of WNV by *Culex* mosquitoes may accelerate sharply with an increase in temperature [34,35,36]. If the values of GP and EIP are experimentally estimated for a certain mosquito vector and WNV strain, the “biological”/“process-driven” models may be constructed to analyze the temperature effects on the spatiotemporal dynamics of WNV transmission [37]. Unfortunately, such data were not available for “Russian” WNV strains and Russian populations of *Culex* mosquitoes, so we had to use purely “statistical” approaches for modeling based on solely human WND incidence. 

Territorial factors like urbanization and population density showed also the effect on WND incidence, as observed in US-North East and Iowa [32,38]. Discriminant analysis of characteristics of regions in the Canadian Saskatchewan province in 2003 and 2007 revealed the relationship between high WND incidence and the following risk factors: low precipitation in June–July and high temperatures in July–August [28]. According to the data obtained in Illinois, the value of cumulative temperatures (above 22 °C) was the best differentiator of years with high WND incidence and high WNV infection rate of *Culex* mosquitoes. Dry spring followed by humid summer also contributed to the infection spread, though to a lesser extent. Areas with high incidence and mosquitoes infection rate were characterized by lesser summer precipitation [29]. During the WND outbreak in Europe in 2010, the human morbidity correlated with the higher temperature and, to a lesser extent, with relative humidity, while the association with precipitation was not consistent. Notably, northern (“colder”) countries displayed strong correlations between a number of WND cases and temperature with a lag of up to four weeks, in contrast to southern (“warmer”) countries, where the response was immediate [5].

Such relations should be used to develop models based on data gathered daily for a large territory through remote satellite monitoring, to forecast the risk of WND outbreaks [39,40]. Data on the timing of spring green up (measured with NDVI), temperature variability in early spring and summer (measured with land surface temperature), and moisture availability from late spring through early summer (measured with actual evapotranspiration) can be useful predictor of the risk of human WND infection, while abundance of mosquitoes may be predicted based on values of daily surface water inundation fraction, surface air temperature, soil moisture, and microwave vegetation opacity.

The current study has been designed to identify climatic and environmental factors that have considerably influenced the WND incidence in endemic regions of southern Russia in recent years, and to make, with these factors taken into account, models that would enable to forecast epidemiological situation of WND in the current year.

## 2. Materials and Methods

Russia’s territory is administratively divided into 83 so-called “constituent entities of the Russian Federation” (province, territory or republic). The data on WND incidence in the Russian Federation constituent entities are based on official statistics of the Ministry of Health (Rospotrebnadzor) and research papers published over the period of 1999–2013 [5,6,12,41,42,43,44,45].

**Figure 1:**
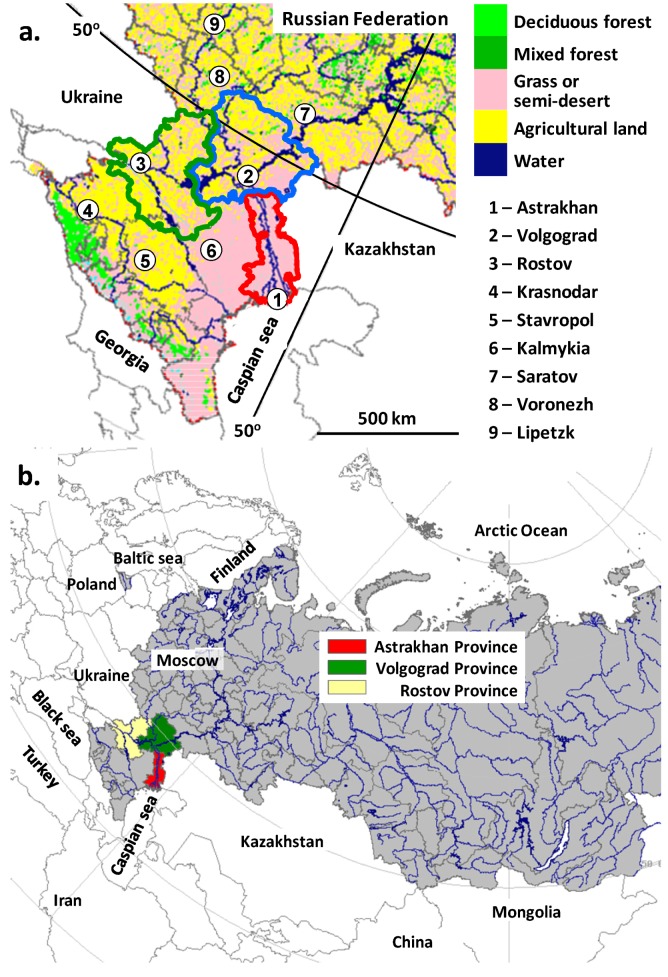
(**a**) Map of Southern European Russia where WND clinical cases have been diagnosed or possible. (**b**) the map of Russia and neighboring countries in polar projection.

The main dataset was made with data of WND occurred from 2001 to 2012 in Astrakhan, Volgograd and Rostov Provinces (Figure 1). These data, that include about 58% of 2,283 WND cases reported in Russia from 1999 to 2013, may be considered informative and reliable since they were gathered using standardized procedures according to official Methodological Guidelines [45,46,47,48].

All patients with high fever or neuroinvasive disease hospitalized in these three provinces from June to October in 2000–2013 underwent laboratory investigation including serological testing. By definition, a WND case was mandatory notified if all other possible diagnoses were excluded and high titer of WNV antibodies was found in a serum samples by IgM-capture ELISA. Up to 30% of WND cases were additionally confirmed by IgG seroconversion and/or specific PCR but these tests were not mandatory. All patients died because encephalitis in this period were subjected to PCR-based investigation and the presence of WNV RNA in a brain autopsy sample was considered as laboratory confirmation of WNV infection. Thus, the case definition included both WND neuroinvasive and non-neuroinvasive cases which were not separately notified [45,46,47]. These procedures could lead to some underestimation of WND incidence, because the patients with mild symptoms were not hospitalized and investigated, or its overestimation because of false-positive ELISA results or the presence of IgM antibody developed in response to a previous WNV exposure. Therefore, we had not used the absolute values of the WND incidence for decision trees. The trees were constructed using the changes of incidence in the province in comparison with the incidence in a previous year (see below).

Incidence in a particular year was compared to factors’ values throughout a “WND epidemic year”, beginning from November of the previous (as relative to the incidence data) year through October of the current year. Two types of models were made—“explanatory” and “prognostic”. Explanatory Models (ExpMod) took into account variations of climatic and environmental parameters over the entire “epidemic year” including the period of the highest WND incidence, from July to October (This period will be called “an epidemic season” below.) Prognostic Models (ProMod) only used parameters’ values from November to June, that is, they were, by definition, intended to “predict” incidence changes before the beginning of an epidemic season.

In order to build the model, the events subjected to analysis and forecasting were classified by three types of outcome: (1) decrease of incidence in the province in comparison with the incidence in the previous year (by at least 15% from the long-term average for the province in question); (2) stabilization of incidence (the value of the current year differs from last year’s value by less than 15% from the long-term average); (3) increase of incidence in the province in comparison with the incidence in a previous year (by at least 15% from the long-term average). The long-term average was defined as the arithmetic mean of WND incidence in 2001–2012. The threshold of 15% was arbitrary.

The incidence in the three provinces is analyzed for correlation with 96 variables averaged for the same entity: mean monthly temperature (T); mean monthly relative humidity (RH); mean monthly atmospheric pressure (AtmP); mean monthly amount of precipitation per day (AP); mean monthly value of NDVI (NDVI); mean monthly value of NDVI for forests (NDVI-forest); mean monthly value of NDVI for meadow and steppe vegetation (NDVI-meadow); mean monthly value of NDVI for arable lands (NDVI-arable). So 8 environmental parameters were considered for each month of “epidemic year”.

Primary meteorological data were freely available from National Center for Atmospheric Research (NCAR, Boulder, CO, USA) [49]. NDVI was calculated from MODIS data using standard product MOD 09 [50,51]. Primary data were re-analyzed, cleared from noise, and linked to Russian administrative regions and cities using techniques of the VEGA geoportal created and maintained by the Space Research Institute of the Russian Academy of Sciences [51,52]. The maps of land cover were also based on MODIS data and produced using original method developed in the Space Research Institute [51,53]. For the purpose of this study the values of climatic and environmental parameters were directly retrieved from a database making the basis for VEGA geoportal.

Statistical analysis was performed with the IBM SPSS Statistics 19 software [54]. The Decision Tree procedure tested the hypothesis of an association between climatic and environmental parameters and increasing, decreasing or stable incidence of WND. The CRT method (Classification and Regression Trees) was used; verification also involved using the CHIAD method (Chi-squared Automatic Interaction Detection). A constructed final tree was crossvalidated dividing the sample into 10 of subsamples. Tree models were then generated, excluding the data from each subsample in turn. The crossvalidated misclassification risk estimate for the final tree was calculated as the average of the risks for all of the trees [55].

The task of the Decision Tree procedure was to select several most significant parameters and thresholds for their categorization so that their combination allowed to classify and “predict” one of three events: decrease, stability, or increase of WND incidence in comparison with a previous year. The term “correct classification” below means that Decision Tree procedure forms a pure terminal node containing only one “correct” type of outcome (e.g., the node with six years of increase in Figure 6a). If a terminal node contains several types of outcome (e.g., the node with one year of stability and four years of decrease in Figure 6a), a prevalent outcome is considered to be correctly classified and less frequent outcomes are formally considered as “errors”.

## 3. Results and Discussion

**Figure 2:**
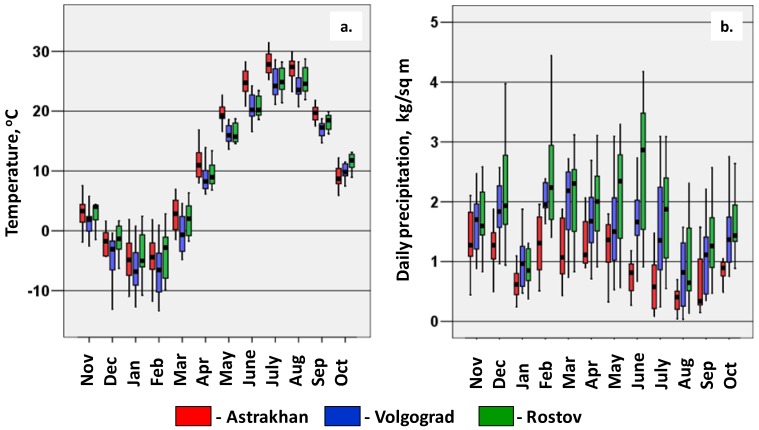
(**a**) Box-and-whisker plot of mean monthly temperature. (**b**) precipitation in Astrakhan, Volgograd and Rostov Provinces in 2001–2012. 1 kg/sq·m corresponds to 1 mm of rainfall.

Figure 1 presents a map of the south of Russia, where most of the WND cases were reported; Figure 2 shows the seasonal changes of temperature and precipitation in Astrakhan, Volgograd and Rostov Provinces with continental climate. In general, winter is colder in Volgograd and spring—summer months are warmer in Astrakhan Province that is also more arid. In Southern Russia there are two large rivers, the Volga and the Don, with their tributaries, artificial lakes and channels, marshland and ponds. As a result a lot of migrating birds from Africa, mainly waterfowl, nest in this region in spring—summer. The capitals, Astrakhan City and Volgograd City are on the banks of the Volga river, and Rostov City is on the banks of the Don river. The land use derived from remote sensing data [50,53] is coded by colors in Figure 1 and shown in Table 1. The urbanization level is moderate. Yellow “agricultural land” in Volgograd and Rostov Provinces means mainly cropland. Pink “grassland, steppe and semi-desert” in Astrakhan and Volgograd Provinces are used for grazing or gardening, or not used. Large forests (“green colors”, Figure 1) are nearly absent although there is a lot of vegetation near rivers and ponds.

**Table 1:** Demographical data, land use and vegetation in Astrakhan, Volgograd and Rostov Provinces, mean values of 2001–2012 years.

	Astrakhan Province	Volgograd Province	Rostov Province
Total population	1,000,000	2,600,000	4,300,000
Proportion of urban residents in the total population, %	67%	76%	67%
Total area, sq km	44,100	113,900	100,800
Agricultural land, cropland, %	1%	38.1%	52.3%
Steppe (grass and/or semi-desert), %	55.2%	29.6%	14.6%
Grassland, %	37.1%	22.4%	23.6%
Soil with a minimum of vegetation, %	5.4%	5.2%	6.2%
Water surface, %	1.0%	2.3%	1.1%
Deciduous forest, %	0.0%	1.2%	0.8%
Light coniferous forest, %	0.0%	0.8%	1.0%
Urban zone, %	0.3%	0.3%	0.4%

**Figure 3:**
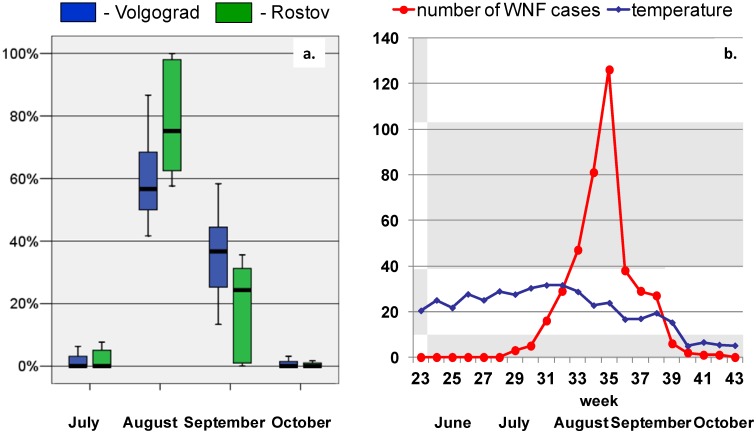
(**a**) Box-and-whisker plot of the number of WND cases per month (in proportion to the number per an year) in Volgograd and Rostov Provinces in the period 2001–2012. (**b**) number of WND cases per week and mean weekly temperature in Volgograd Province in 2010.

In total 1,335 WND cases were reported during the study period (2001–2012): 350, 798 and 187 cases in Astrakhan, Volgograd and Rostov Provinces, respectively. Official Russian registries do not discriminate non-neuroinvasive and neuroinvasive forms of WND. Apparently, neuroinvasive cases amounted to less than 10% [41,42,43,44,45]. The overall case-fatality rate was about 2%. The ratio of WND incidence in rural and in urban population was 0.40 and 0.35 (median values) for Volgograd and Rostov Provinces, respectively. This ratio was significantly higher (1.1) in Astrakhan Province (*p* < 0.05). Most WND cases occurred in August-September (Figure 3a). The incidence peaks usually at weeks 34–36 (August 19—September 9) (Figure 3b).

Table 2 presents the WND incidence in Astrakhan, Volgograd and Rostov Provinces over the period from 1997 to 2013. The data on WND cases in other Russian provinces in 2010–2013 can be found in the ECDC website [6].

According to Table 2, in the three provinces different WND epidemiological situations have been observed from year to year during the study period. Major epidemics occurred in 2012 in all the three provinces, in 1999 in Astrakhan and Volgograd, in 2010 in Volgograd and Rostov, and in 2005 in Astrakhan. No cases were recorded from 1997 to 1999 and in 2002 in Rostov Province, and in 2003–2004 in Volgograd Province, while in Astrakhan the lowest incidence (0.1 cases/100,000) was observed in 2008.

**Figure 4:**
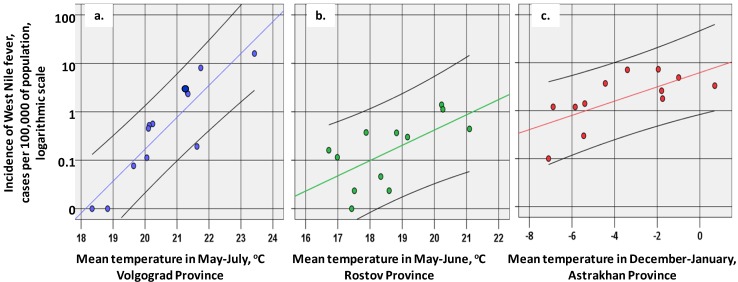
(**a**) Scatterplots of WND incidence in 2001–2012 *vs.* mean temperature in May—July in Volgograd Province. (**b**) mean temperature in May-June in Rostov Province. (**c**) mean temperature in December—January in Astrakhan Province.

Correlations between mean temperatures and the incidence are presented in Table 3 and in Figure 4. In some cases the average temperature for several months, e.g., December–January, correlated better with WND incidence than any single monthly temperature (the average December–January T is defined as (“December T” + “January T”)/2.) It is noteworthy that other factors, namely precipitation, relative humidity, atmospheric pressure or NDVI values, did not significantly correlate with the WND incidence in any of the provinces.

**Table 2:** Absolute values and trends of WND incidence in Astrakhan, Volgograd, and Rostov Provinces, Southern Russia, from 1997 to 2013.

	Astrakhan Province		Volgograd Province		Rostov Province	
Year	WND incidence per 100,000 population	Classifi-cation of changes in WND incidence	WND incidence per 100,000 population	Classifi-cation of changes in WND incidence	WND incidence per 100,000 population	Classifi-cation of changes in WND incidence
1997	0.80		> 0.19		0?	
1998	0.90	Stability *	> 1.31	Increase?	0?	
1999	**9.50**	Increase	**> 14.3**	Increase	0?	
2000	3.70	Decrease	1.20	Decrease	0.11	Increase?
2001	4.90	Increase	0.56	Decrease	0.11	Stability
2002	3.70	Decrease	0.53	Stability	0.00	Decrease
2003	1.20	Decrease	0.00	Decrease	0.05	Increase
2004	2.60	Increase	0.00	Stability	0.16	Increase
2005	**7.30**	Increase	0.11	Stability	0.37	Increase
2006	1.41	Decrease	0.45	Stability	0.30	Decrease
2007	3.32	Increase	2.40	Increase	0.44	Increase
2008	0.10	Decrease	0.08	Decrease	0.02	Decrease
2009	0.30	Stability	0.19	Stability	0.02	Stability
2010	1.19	Increase	**15.92**	Increase	**1.39**	Increase
2011	1.78	Increase	2.34	Decrease	0.37	Decrease
2012	**7.11**	Increase	**8.07**	Increase	**1.12**	Increase
2013	6.03	Decrease	1.85	Decrease	0.19	Decrease
Mean, 2001–2012	2.91		2.55		0.36	

* The rules for classification of WND incidence changes as “decrease”, “stability” and “increase” are given in the Methods; ? The data obtained from official publications were considered not reliable and were not used below.

For Volgograd Province, the most important relation was the correlation of morbidity with May to July temperatures; so WND outbreaks occurred in the years, when the mean temperature in May—July exceeded 21 °C (four points in the upper right corner of Figure 4a corresponding to years 2007 and 2010–2012). As a linear approximation (Figure 4a) suggests, on average, a 1 °C rise of temperature at this period would cause an increase of incidence with a factor of 4.6.

In Rostov Province, the most important factor was the temperature in May, and (to a lesser extent) in June, but not in July. Incidence increases were also associated with warmer than usual temperatures in December of the previous year. In the southernmost Astrakhan Province, summer temperatures are generally rather high (Figure 3a) and do not restrict the spread of the WND. In this province, the temperature of December—January may act as a limiting factor: when below −5 °C, WND incidence tends to be lower than usual (five points in the lower left corner of Figure 4c corresponding to years 2003, 2006, and 2008–2010). In all the three provinces, a weak positive correlation was observed with temperatures in August—September, when most of the WND cases were recorded (Figure 2a, Figure 3). Owing to a relatively short observation period, this correlation did the statistically significant value (*p* = 0.05).

Linear fit lines are colored, black lines shows 95% confidence intervals for individual values.

**Table 3:** Correlation between temperature characteristics and WND incidence in three Russian Provinces in 2001–2012.

Province:	Astrakhan	Volgograd	Rostov
**Mean temperature in one or several months**	Spearman’s correlation coefficient and significance level (*p* value)		
December	0.31 (0.32)	0.47 (0.12)	**0.64 (0.026) ^1^**
January	**0.66 (0.020)**	−0.014 (0.96)	0.23 (0.47)
May	0.38 (0.23)	0.44 (0.15)	**0.71 (0.010)**
June	−0.18 (0.57)	**0.64 (0.024)**	0.50 (0.095)
July	−0.042 (0.90)	**0.65 (0.023)**	−0.007 (0.98)
August	−0.007 (0.98)	0.43 (0.16)	0.46 (0.13)
September	0.40 (0.20)	0.31 (0.33)	0.32 (0.32)
December—January	**0.70 (0.011)**	0.24 (0.46)	0.47 (0.12)
May—June	0.084 (0.80)	**0.62 (0.031)**	**0.69 (0.014)**
May—July	0.18 (0.59)	**0.89 (<0.001)**	0.48 (0.12)
August—September	0.25 (0.44)	0.42 (0.17)	0.41 (0.18)

^1^ In bold the correlation coefficients greater than zero with *p* < 0.05. Results for November, February, March, April and October are omitted because not statistically significant.

**Figure 5:**
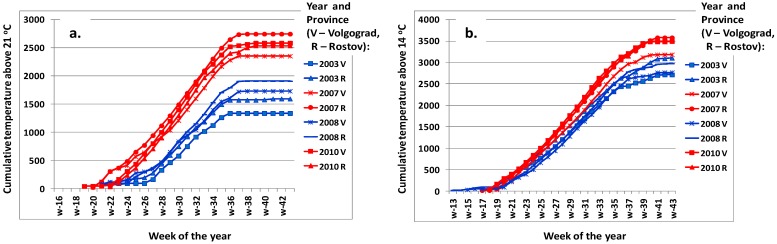
(**a**) Weekly cumulative temperature values above a threshold 21 °C and 14 °C. (**b**) measured in Volgograd and Rostov Provinces, Southern Russia. Years with low and high WND incidence are in blue and red, respectively.

In Figure 5 cumulative spring-summer temperatures in Volgograd and Rostov Provinces in years with low WND incidence (2003 and 2008) and WND outbreaks (2007 and 2010) are plotted. In years with low incidence, the period, when the mean temperatures steadily exceed 21 °C, come a few weeks later, and the final cumulative temperatures are lower. The effect of temperatures above the threshold value of 14 °C is less obvious.

**Figure 6:**
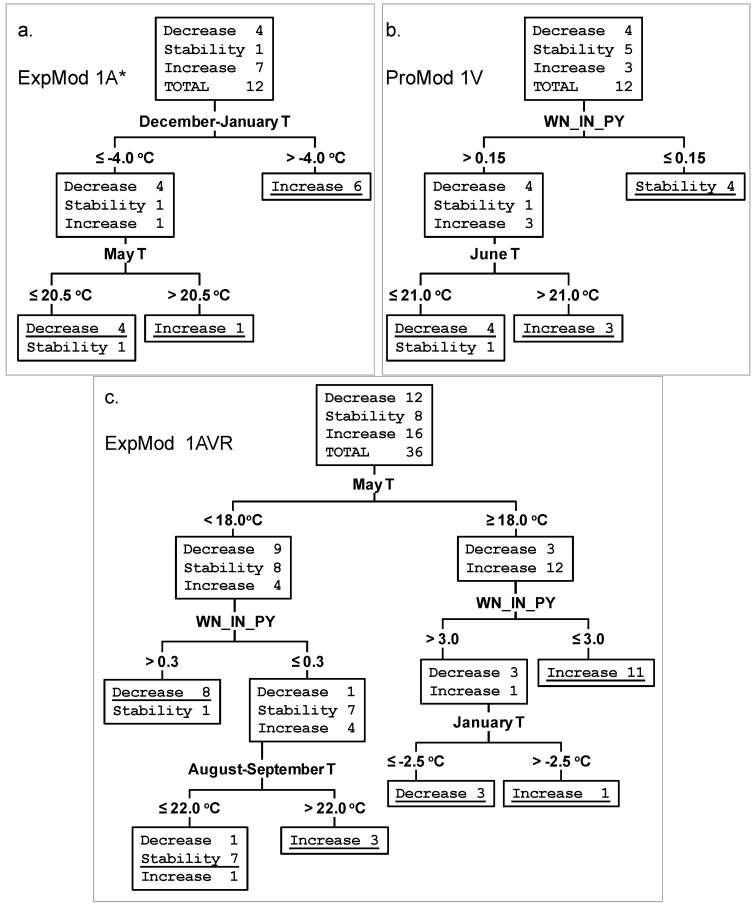
Decision trees showing three examples of classification algorithms for years with decreasing, stable, and increasing WND incidence. The parameters and their threshold values are indicated on the branches. Correct classification in terminal nodes is underlined. (**a**) ExpMod 1A* for Astrakhan Province. (**b**) ProMod 1V for Volgograd Province. (**c**) ExpMod 1AVR* for three provinces of Southern Russia together.

For planning and implementation of preventive measures and control of epidemics, it is necessary, above all, to know and predict the pattern of the incidence variation from information obtained in the previous year(s). ExpMod and, particularly, ProMod were designed for this purpose. Only main dataset, that is the data relating to years 2001-2012, were used to build these models. In addition to mean monthly parameters, we included also the average temperature of different time periods of the year that showed significant correlations with WND incidence (Table 3). Taking into account that the WNV activity in the previous year might modulate the WND morbidity in the current year, we have also added the variable “WND incidence in the previous year” as the independent “predicting” variable. This variable is designated as WN_IN_PY below. With regard the ExpMod and ProMod, only the models capable to provide the better explanation (ExpMod) or prediction (ProMod) for each province with the minimum number of parameters are presented. One of these models was constructed without variable WN_IN_PY and second model included the variable WN_IN_PY. We have also plotted the model common for all the three provinces, though their geographical location, fauna, type of landscape and other characteristics are not identical, which could have modified the effects of climatic and environmental factors being assessed. The logical structure, thresholds and performance of seven selected models is presented in Table 4. Decision trees corresponding to three of the seven models are shown in Figure 6.

**Table 4:** Structure and performance of the selected models.

Province	Type of model	Value of parameters and/or their combinations	Prediction of model and number of years	Real events in these years	% of correct classifi-cations
**Astrakhan**	ExpMod 1A *	December—January T > −4.0 °C	Increase for 6 years	Increase in 6 years	100%
		December-January T ≤ −4.0 °C and May T ≥ 20.5 °C	Increase for 1 year	Increase in 1 year	100%
		December-January T ≤ −4.0 °C and May T < 20.5 °C	Decrease for 5 years	Decrease in 4 years, *stability in 1 year* **	80%
		**Total % of correct classifications for ExpMod 1A**			**92%**
**Astrakhan**	ExpMod 2A *	WN_IN_PY *** < 3.0 and December T > −4.0 °C	Increase for 6 years	Increase in 6 years	100%
		WN_IN_PY < 3.0 and December T < −4.0 °C	Stability for 1 year	Stability in 1 year	100%
		WN_IN_PY > 3.0 and January T > −2.0 °C	Increase for 1 year	Increase in 1 year	100%
		WN_IN_PY > 3.0 and January T < −2.0 °C	Decrease for 4 years	Decrease in 4 years	100%
		**Total % of correct classifications for ExpMod 2A**			**100%**
**Volgograd**	**ExpMod 1V**	May—June T > 19.5 °C	Increase for 3 years	Increase for 3 years	100%
		May—June T ≤ 19.5 °C and September T > 16.5 °C	Stability for 5 year	Stability in 5 year	100%
		May—June T ≤ 19.5 °C and September T ≤ 16.5 °C	Decrease for 4 years	Decrease in 4 years	100%
		**Total % of correct classifications for ExpMod 1V**			**100%**
**Volgograd**	**ProMod 1V**	WN_IN_PY > 0.15 and June T > 21.0 °C	Increase for 3 years	Increase in 3 years	100%
		WN_IN_PY < 0.15	Stability for 4 year	Stability in 4 year	100%
		WN_IN_PY > 0.15 and June T < 21.0 °C	Decrease for 5 years	Decrease in 4 years, *stability in 1 year* **	80%
		**Total % of correct classifications ProMod 1V**			**92%**
**Rostov**	ExpMod 1R *	May T > 16.5 °C	Increase for 5 years	Increase in 5 years	100%
		May T < 16.5 °C and January T > −3.0 °C	Increase for 2 years	Increase in 1 years, stability in 1 year **	50%
		May T < 16.5 °C and January T ≤ −3.0 °C	Decrease for 5 years	Decrease in 4 years, stability in 1 year **	80%
		**Total % of correct classifications ExpMod 1R**			**83%**
**Rostov**	ExpMod 2R *	WN_IN_PY < 0.40 and May T > 16.5 °C	Increase for 5 years	Increase in 5 years	100%
		WN_IN_PY < 0.40 and May T < 16.5 °C and December—January T > −2.0 °C	Increase for 2 years	Increase in 1 years, stability in 1 year **	50%
		WN_IN_PY < 0.40 and May T < 16.5 °C and December—January T < −2.0 °C	Decrease for 3 years	Decrease in 2 years, stability in 1 year **	67%
		WN_IN_PY > 0.40	Decrease for 2 years	Decrease in 2 years	100%
		**Total % of correct classifications ExpMod 2R**			**83%**
**Astrakhan Volgograd Rostov**	**ExpMod 1AVR**	May T ≥ 18.0 °C and WN_IN_PY ≤ 2.5	Increase for 11 years	Increase in 11 years	100%
		May T ≥ 18.0 °C and WN_IN_PY > 3.0 and January T > −2.5 °C	Increase for 1 year	Increase in 1 year	100%
		May T ≥ 18.0 °C and WN_IN_PY > 3.0 and January T < −2.5 °C	Decrease for 2 years	Decrease in 2 years	100%
		May T < 18.0 °C and WN_IN_PY ≤ 0.3 and August-September T > 22 °C	Increase for 3 years	Increase in 3 years	100%
		May T < 18.0 °C and WN_IN_PY ≤ 0.3 and August-September T < 22 °C	Stability for 9 years	Stability in 7 years, *increase in 1 year, decrease in 1 year*	78%
		May T < 18.0 °C and WN_IN_PY > 0.3	Decrease for 9 years	Decrease in 8 years, *stability in 1 year*	89%
		**Total % of correct classifications ExpMod 1AVR**			**92%**

* This model was also the best ProMod, as it has been developed using only parameters obtained before the beginning of the “epidemic season” (July-October current year); ** Less frequent outcomes shown in italics are formally considered as “errors of prediction”; *** WN_IN_PY means “WND incidence in the previous year”, no. of cases per 100,000 population.

**Figure 7:**
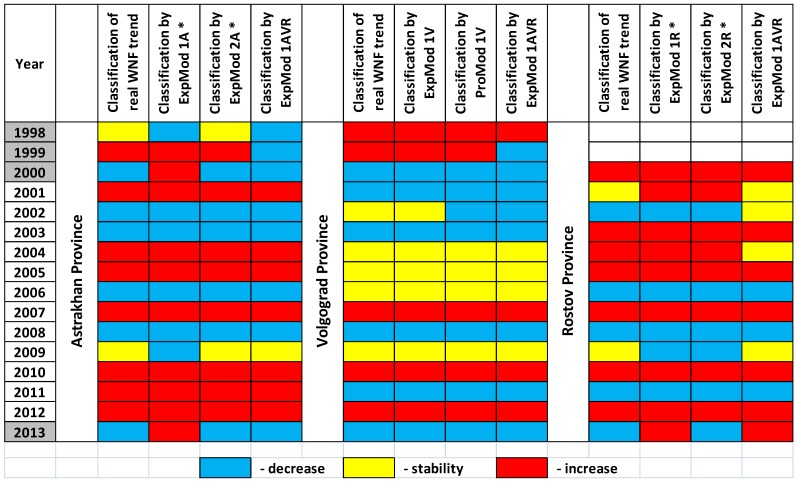
The ability of seven selected models to explain or “predict” the trend of human WND incidence in Astrakhan, Volgograd, and Rostov Provinces. The model were constructed using the data of 2001–2012 and checked using the data of 1998–2000 and 2013.

The best ExpMod 2A* for Astrakhan Province uses three parameters: first of all, the variable WN_IN_PY and then the mean monthly temperatures (T) in December and January (Table 4). Noteworthy, this model was also the best ProMod, as it has been developed using parameters obtained (December previous year and January current year) before the beginning of the epidemic season (July–October current year). ExpMod 2A* classified correctly all the 12 outcomes in 2001–2012 (Figure 7). High WND incidence in the previous year correlates with the decrease of WND incidence in current year. Relatively warm December and January contribute to the increased WND incidence in the following summer. If the variable WN_IN_PY is deliberately excluded from the analysis, the best ExpMod 1A*, which is also ProMod as indicated by an asterisk, uses December–January T and then May T (Table 4 and Figure 6a).

The best ExpMod 1V for Volgograd Province uses May—June T and then September T, classifying correctly all the 12 outcomes in 2001–2012 (Table 4, Figure 7). The increase is expected in case of warm May—June; the decrease is expected if both May—June and September are cold. If the variable WN_IN_PY is included in the analysis, the decision tree procedure selects two variables, WN_IN_PY and June T, and constructs ProMod 1V providing the best “predictions” for 2001–2012 (Table 4, Figure 6b and Figure 7).

For Rostov Province, the best ExpMod 1R* was the one that fitted two parameters, the mean monthly May T and January T. Values of T in May above 16.5 °C were unambiguously related to an increase in the WND incidence in July-October (epidemic season) of the same year. If both May and January were cold, the decrease was predicted (Table 4, Figure 7). ExpMod 2R* took also WN_IN_PY into account. Again the decrease of WND incidence followed years with relatively high WND incidence. ExpMod 1R* and ExpMod 2R* could not correctly classify two years of stability, 2001 and 2009.

The analysis proves that it is possible to build acceptable models which are common for all the three provinces. ExpMod 1AVR provides the best possible classification using temperatures in May, January, and August-September, as well as the variable WN_IN_PY (Table 4, Figure 6c and Figure 7). ExpMod 1AVR makes 3 classification errors (Volgograd Province in 2002, Rostov Province in 2002 and 2004).

It has to be pointed out that including in our models a larger number of parameters would have avoided even these minor classification errors, but we deliberately limited the number of parameters to the 2–4 most important, to prevent the “overfitting” due to the small number of observations. For this purpose, the maximum number of levels for the CRT Growing Method was custom fixed as 3, but many branches of the decision trees were even shorter, having just one or two nodes (Figure 6).

Over the period of 2001–2012, there was an epidemic increase of WND in 2007, 2010, 2012 in all the provinces under consideration, and in 2001, 2004, 2005 and 2011 in Astrakhan only. The adequacy and usefulness of the models is supported by the fact that all 7 selected models were able to explain those increases in all the 13 (3 × 3 + 4) cases. Similarly, five constructed ProMod (ExpMod 1A*, ExpMod 2A*, ProMod 1V, ExpMod 1R*, and ExpMod 2R*) using climatic parameters of November–June were able to “forecast” those increases. 

In general, all seven selected models provide correct classifications for the years 2003, 2005–2008, and 2010–2012. Some, but not all models may not explain the situation in Volgograd in 2002, in Astrakhan in 2009, and in Rostov in 2001, 2002, 2004, and 2009. Notably, these were years of low WND incidence, since only 14, 3, 5, 0, 7, and 1 human WND cases were registered, respectively. They account for only 2% of the WND cases (30 of 1,330) identified in these provinces during the study period 2001–2012. 

With certain caution, the suggested models may be used for analysis of WND incidence in other years, as well. When referred to earlier observations, province-specific ExpMod and ProMod explain and “forecast” an increase in WND incidence in Astrakhan Province in 1999, in Volgograd Province in 1998 and 1999, in Rostov Province in 2000 (Figure 7). For the 2013, we observed conflicting results; the province-specific models ExpMod 1A* and ExpMod 1R* without variable WN_IN_PY “expected” the increase of human WND incidence in Astrakhan and Rostov because of relatively mild winter and warm May. Conversely, province-specific models ExpMod 2A*, ProMod 1V, and ExpMod 1R*, using the variable WN_IN_PY, predicted correctly the decrease of human WND incidence in all three provinces, since human morbidity and, presumably, WNV transmission, had been high in 2012. Noteworthy, major WND outbreaks in Romania, Israel, Greece and Russia have been invariably followed by a relative decline of incidence in the following year [3,4,7,12,13,14,15,20,41,42,43,44,45].

An additional pitfall for modeling was that the WNV strains were not the same in the study period in the three provinces. It can be reasonably assumed that in recent history (*i.e.*, since 1990) there were two independent introductions of WNV strains in Volgograd, possibly by migratory birds from Africa. The first WNV lineage 1a clone (prototype strain VLG-4 AF317203) was introduced around 1995 [9,56]. From 1999 to 2003, all WNV isolates from Volgograd were very similar [3,12,19]. This implies the persistence/overwintering of the WNV clone in Volgograd, while multiple invasions of the same strain appear to be unlikely. Unfortunately, the mechanisms of WNV overwintering in Volgograd have not been properly studied and remain undefined. In 2004, there was no human case of WND in Volgograd, and we were unable to find any WNV RNA in birds or mosquitoes [12]. The climatic conditions in 2003 and 2004 were unfavorable for WNV infection; as a consequence WNV of lineage 1a disappeared and was not found in Volgograd anymore. The second WNV clone, belonging to lineage 2 (prototype strain Reb_VLG_07 FJ425721), was introduced in Russia and Eastern Europe after the year 2000 [12,15,16]. Between 2007 and 2012, all WNV isolates from Volgograd and Rostov were nearly identical to the strain Reb_VLG_07. This implies again the persistence of the WNV clone in Southern Russia. The data on WNV genotypes in Rostov and Astrakhan Provinces are scarce. Apparently, several WNV clones may persist at the same time in more southern Astrakhan Province [12,56,57]. Climatic conditions might facilitate or impede WNV introduction into the northern regions and, vice versa, the features of prevalent WNV strains, for example their virulence for vectors and hosts, might affect WNV transmission independently of climatic factors. Despite that, the same, or similar, models proved to be applicable for the whole study period in all three provinces.

It is noteworthy that relative humidity or atmospheric pressure values were not taken into account in order to explain variations in WND incidence. The increase of incidence is mostly related to higher than normal mean temperatures in May and/or June (the period of WNV amplification in epizootic cycles bird-mosquito-bird) and, to a lesser extent, high temperatures in August–September (the peak of human morbidity). A decline of incidence may be associated with cold winter (December and/or January and/or February, depending on the region and the type of model).

Neither the temperature or precipitation registered in the other months, nor the values of NDVI of various types of vegetation, appeared to produce an independent statistically significant effect on the WND incidence, as their potential positive or negative effects were weaker than the effect produced by significant factors.

Our “statistical” findings could not be automatically translated into the language of process-driven models. Biological mechanisms affected by warm temperature include the shortening of the GP duration of the mosquito and the EIP of the virus both of which increase efficiency of WNV transmission [34,35,36,37]. The variations of air temperature in the range from 14 °C to 30 °C are critical and this is exactly the range of temperatures observed in Southern European Russia in May-September (Figure 2a). Unfortunately, Russian data on WNV vectors and hosts are scarce. WNV strains present in Astrakhan, Volgograd and Rostov has not been found outside Russia and Romania, and the populations of main Russian WNV vectors, *Cx. pipiens pipiens*, *Cx. pipiens molestus*, and *Cx. modestus* have some genetic and ecological differences from European and American populations [3,21]. Thus, the exact relation of the GP and EIP with the temperature is not known for these vectors and WNV strains, although our field studies in Volgograd showed that *Culex* mosquitoes abundance in an epidemic season was higher in the years with a mild winter and a hot summer [3].

To our knowledge, the effect of winter temperatures below zero has not been previously noted. In contrast to most WNV endemic regions, winters in Russian continental climate are really cold, with temperature that can reach −30°C (Figure 2a), that probably affects the survival of overwintering mosquitoes outdoors (both Culex imago and Aedes eggs) [3,23]. The development of autogenous *C. pipiens* overwintering in non-heated basements may be also affected by ambient temperature. 

In general, the effects of precipitation on WNV transmission and human WBF morbidity remains controversial [5,28,29,32,33]. Apparently, in contrast to the more arid regions [58], the amount of rainfall in the epidemic season is not essential in Southern Russia. The influence of relative humidity and precipitation in the epidemic season is minimized, that is probably due to the fact that in most cases, WND in Russia is observed near major rivers (the Volga, the Don), artificial water reservoirs, lakes, or marshlands. In these areas the mosquito can find suitable places for breeding and survival irrespective of the amount of precipitation.

Our analysis suggests that the weather effects are most critical in temperate climate at the northern border of WND area, for example in Volgograd, where the amplitude of fluctuation of human WND incidence is huge (Table 2). However, the cyclic changes in the WND incidence may be partly due to the natural cyclic changes of avian host immunity [27].

## 4. Conclusions

The results of this study confirm and ascertain our preliminary hypotheses [2,3] and, with Russia’s geographical, ecological, climatic and epidemiological peculiarities taken into account, are close to results of Paz *et al*. [5] reporting that “For human morbidity, significant positive correlations were observed between a number of WND cases and temperature, with a geographic latitude gradient: northern (“colder”) countries displayed strong correlations with a lag of up to four weeks, in contrast to southern (“warmer”) countries, where the response was immediate”. In Russia, like Romania, and differently to what observed in the southern countries of Greece, the Balkans or Italy, the first human cases of WND are recorded from mid-July and the incidence peaks is in late August–early September. However, the incidence is more affected by higher temperatures in May to June. In Russia’s continental climate, winter weather conditions are also important since low temperatures in winter are related to lower WND incidence in the subsequent year. The WND incidence also tended to decrease after the years of major WND outbreaks, probably, because of the effects of previously acquired avian herd immunity. On the whole, there is, beyond doubt, a dependence of WND epidemiological pattern on climatic and weather factors, which enables to develop and (along with accumulation of observation data) ascertain models predicting the changes in the risk of this emerging infection.

## References

[1] Hayes E.B., Sejvar J.J., Zaki S.R., Lanciotti R.S., Bode A.V., Campbell G.L. (2005). Virology, pathology, and clinical manifestations of West Nile virus disease. Emerg. Infect. Dis..

[2] Platonov A.E. (2006). The influence of weather conditions on the epidemiology of vector-borne diseases by the example of West Nile fever in Russia. Vestn. Ross. Akad. Med. Nauk..

[3] Platonov A.E., Fedorova M.V., Karan L.S., Shopenskaya T.A., Platonova O.V., Zhuravlev V.I. (2008). Epidemiology of West Nile infection in Volgograd, Russia, in relation to climate change and mosquito (Diptera: Culicidae) bionomics. Parasitol. Res..

[4] Gubler D.J. (2007). The continuing spread of West Nile virus in the western hemisphere. Clin. Infect. Dis..

[5] Paz S., Malkinson D., Green M.S., Tsioni G., Papa A., Danis K., Sirbu A., Ceianu C., Katalin K., Ferenczi E. (2013). Permissive summer temperatures of the 2010 European West Nile fever upsurge. PLoS One.

[6] European Centre for Disease Prevention and Control. http://ecdc.europa.eu/en/healthtopics/west_nile_fever/West-Nile-fever-maps/Pages/index.aspx.

[7] Zeller H.G., Schuffenecker I. (2004). West Nile virus: An overview of its spread in Europe and the Mediterranean basin in contrast to its spread in the Americas. Eur. J. Clin. Microbiol. Infect. Dis..

[8] Artsob H., Gubler D.J., Enria D.A., Morales M.A., Pupo M., Bunning M.L., Dudley J.P. (2009). West Nile Virus in the New World: Trends in the spread and proliferation of West Nile Virus in the Western Hemisphere. Zoonoses Public Health.

[9] May F.J., Davis C.T., Tesh R.B., Barrett A.D. (2011). Phylogeography of West Nile virus: From the cradle of evolution in Africa to Eurasia, Australia, and the Americas. J. Virol..

[10] Bakonyi T., Ivanics E., Erdelyi K., Ursu K., Ferenczi E., Weissenbock H., Nowotny N. (2006). Lineage 1 and 2 strains of encephalitic West Nile virus, central Europe. Emerg. Infect. Dis..

[11] Shopenskaya T.A., Fedorova M.V., Karan L.S., Frolov A.Y., Malenko G.V., Levina L.S., Pogodina V.V., Platonov A.E. (2008). New variant of West Nile virus and its potential epizootic and epidemic importance. Epidemiol. Infect. Dis..

[12] Platonov A.E., Karan L.S., Shopenskaia T.A., Fedorova M.V., Koliasnikova N.M., Rusakova N.M., Shishkina L.V., Arshba T.E., Zhuravlev V.I., Govorukhina M.V. (2011). Genotyping of West Nile fever virus strains circulating in Southern Russia as an epidemiological investigation method: Principles and results. Zh. Mikrobiol. Epidemiol. Immunobiol..

[13] Danis K., Papa A., Theocharopoulos G., Dougas G., Athanasiou M., Detsis M., Baka A., Lytras T., Mellou K., Bonovas S., Panagiotopoulos T. (2011). Outbreak of West Nile virus infection in Greece, 2010. Emerg. Infect. Dis..

[14] Kalaycioglu H., Korukluoglu G., Ozkul A., Oncul O., Tosun S., Karabay O., Gozalan A., Uyar Y., Caglayık D.Y., Atasoylu G. (2012). Emergence of West Nile virus infections in humans in Turkey, 2010 to 2011. Euro Surveill..

[15] Neghina A.M., Neghina R. (2011). Reemergence of human infections with West Nile virus in Romania, 2010: An epidemiological study and brief review of the past situation. Vector Borne Zoonotic Dis..

[16] Ciccozzi M., Peletto S., Cella E., Giovanetti M., Lai A., Gabanelli E., Acutis P.L., Modesto P., Rezza G., Platonov A.E., Lo Presti A., Zehender G. (2013). Epidemiological history and phylogeography of West Nile virus lineage 2. Infect. Genet. Evol..

[17] Hayes E.B., Komar N., Nasci R.S., Montgomery S.P., O’Leary D.R., Campbell G.L. (2005). Epidemiology and transmission dynamics of West Nile virus disease. Emerg. Infect. Dis..

[18] Hamer G.L., Kitron U.D., Brawn J.D., Loss S.R., Ruiz M.O., Goldberg T.L., Walker E.D. (2008). *Culex pipiens* (Diptera: Culicidae): A bridge vector of West Nile virus to humans. J. Med. Entomol..

[19] Platonov A.E. (2001). West Nile encephalitis in Russia 1999–2001: Were we ready? Are we ready?. Ann. N. Y. Acad. Sci..

[20] Murgue B., Zeller H., Deubel V. (2002). The ecology and epidemiology of West Nile virus in Africa, Europe and Asia. Curr. Top. Microbiol. Immunol..

[21] Fyodorova M.V., Savage H.M., Lopatina J.V., Bulgakova T.A., Ivanitsky A.V., Platonova O.V., Platonov A.E. (2006). Evaluation of potential West Nile virus vectors in Volgograd region, Russia, 2003 (Diptera: Culicidae): Species composition, bloodmeal host utilization, and virus infection rates of mosquitoes. J. Med. Entomol..

[22] Turell M.J., Mores C.N., Dohm D.J., Komilov N., Paragas J., Lee J.S., Shermuhemedova D., Endy T.P., Kodirov A., Khodjaev S. (2006). Laboratory transmission of Japanese encephalitis and West Nile viruses by molestus form of *Culex pipiens* (Diptera: Culicidae) collected in Uzbekistan in 2004. J. Med. Entomol..

[23] Andreadis T.G., Armstrong P.M., Bajwa W.I. (2010). Studies on hibernating populations of Culex pipiens from a West Nile virus endemic focus in New York City: parity rates and isolation of West Nile virus. J. Amer. Mosquito Contr. Assn..

[24] Gubler D.J., Reiter P., Ebi K.L., Yap W., Nasci R., Patz J.A. (2001). Climate variability and change in the United States: Potential impacts on vector- and rodent-borne diseases. Environ. Health Perspect..

[25] Paz S., Albersheim I. (2008). Influence of warming tendency on Culex pipiens population abundance and on the probability of West Nile fever outbreaks (Israeli Case Study: 2001–2005). Ecohealth.

[26] Semenza J.C., Suk J.E., Estevez V., Ebi K.L., Lindgren E. (2012). Mapping climate change vulnerabilities to infectious diseases in Europe. Environ. Health Perspect..

[27] Kwan J.L., Kluh S., Reisen W.K. (2012). Antecedent avian immunity limits tangential transmission of West Nile virus to humans. PLoS One.

[28] Epp T.Y., Waldner C.L., Berke O. (2009). Predicting geographical human risk of West Nile virus—Saskatchewan, 2003 and 2007. Can. J. Public Health.

[29] Ruiz M.O., Chaves L.F., Hamer G.L., Sun T., Brown W.M., Walker E.D., Haramis L., Goldberg T.L., Kitron U.D. (2010). Local impact of temperature and precipitation on West Nile virus infection in Culex species mosquitoes in northeast Illinois, USA. Parasit. Vectors..

[30] Kilpatrick A.M., Pape W.J. (2013). Predicting human West Nile virus infections with mosquito surveillance data. Amer. J. Epidemiol..

[31] Wang J., Ogden N.H., Zhu H. (2011). The impact of weather conditions on *Culex pipiens* and *Culex restuans* (Diptera: Culicidae) abundance: A case study in Peel Region. J. Med. Entomol..

[32] de Groote J.P., Sugumaran R., Brend S.M., Tucker B.J., Bartholomay L.C. (2008). Landscape, demographic, entomological, and climatic associations with human disease incidence of West Nile virus in the state of Iowa, USA. Int. J. Health Geogr..

[33] Soverow J.E., Wellenius G.A., Fisman D.N., Mittleman M.A. (2009). Infectious disease in a warming world: how weather influenced West Nile virus in the United States (2001–2005). Environ. Health Perspect..

[34] Kilpatrick A.M., Meola M.A., Moudy R.M., Kramer L.D. (2008). Temperature, viral genetics, and the transmission of West Nile virus by *Culex pipiens* mosquitoes. PLoS Pathog..

[35] Richards S.L., Lord C.C., Pesko K.N., Tabachnick W.J. (2010). Environmental and biological factors influencing *Culex pipiens quinquefasciatus* (Diptera: Culicidae) vector competence for West Nile Virus. Amer. J. Trop. Med. Hyg..

[36] Reisen W.K., Fang Y., Martinez V.M. (2006). Effects of temperature on the transmission of West Nile virus by *Culex tarsalis* (Diptera: Culicidae). J. Med. Entomol..

[37] Hartley D.M., Barker C.M., Le Menach A., Niu T., Gaff H.D., Reisen W.K. (2012). Effects of temperature on emergence and seasonality of West Nile virus in California. Amer. J. Trop. Med. Hyg..

[38] Brown H.E., Childs J.E., Diuk-Wasser M.A., Fish D. (2008). Ecological factors associated with West Nile virus transmission, northeastern United States. Emerg. Infect. Dis..

[39] Chuang T.W., Wimberly M.C. (2012). Remote sensing of climatic anomalies and West Nile virus incidence in the Northern Great Plains of the United States. PLoS One.

[40] Chuang T.W., Henebry G.M., Kimball J.S., Vanroekel-Patton D.L., Hildreth M.B., Wimberly M.C. (2012). Satellite microwave remote sensing for environmental modeling of mosquito population dynamics. Remote Sens. Environ..

[41] Onishchenko G.G., Lipnitskii A.V., Alekseev V.V., Antonov V.A., Kriuchkova T.P., Krutogolovova T.A. (2011). Epidemiological situation of West Nile fever in Russia in 2010. Zh. Mikrobiol. Epidemiol. Immunobiol..

[42] Manankov V.V., Alekseev V.V., Smelianskii V.P., Pashanina T.T., Pogasii N.I., Putintseva E.V., Britanova A.L., Antonov V.A., Alekseeva V.V., Tkachenko G.A. (2011). Study of a trend in the epidemic process of West Nile fever in the Volgograd region over the period 2000–2009. Epidemiol. Infect. Dis..

[43] Antonov V.A., Smolenskii V.Iu., Putintseva E.V., Lipnitskii A.V., Smelianskii V.P., Iakovlev A.T., Manankov V.V., Pogasii N.I., Krasovskaia T.I. (2012). West Nile fever epidemic situation in the Russian Federation territory in 2011 and the prognosis of its development. Prob. Especially Dangerous Infect..

[44] Putintseva E.V., Antonov V.A., Viktorov D.V., Smelianskii V.P., Zhukov K.V., Manankov V.V., Pogasii N.I., Tkachenko G.A., Shpak I.M., Snatenkov E.A. (2013). Peculiarities of epidemiological situation on the West Nile fever in 2012 in the territory of the Russian Federation. Prob. Especially Dangerous Infect..

[45] Onishchenko G.G. (2011). The Collection of Materials on the West Nile Fever Outbreak in Russian Federation in 2010.

[46] (2010). Measures against West Nile Fever on the Territory of the Russian Federation. Methodical Guidelines MUK 3.1.3.2600-10.

[47] (2012). The Organization and Execution of West Nile Fever Laboratory Diagnostics in Laboratories at Territorial, Regional and Federal Levels. Methodical Guidelines MUK 4.2.3009-12.

[48] McNamara T., Platonov A., Elleman T., Gresham L. (2013). The human-animal interface and zoonotic threats: The Russian Federation approach. Biosecur. Bioterror..

[49] CISL Research Data Archive. http://rda.ucar.edu.

[50] MOD 09—Surface Reflectance. Atmospheric Correction Algorithm Products. http://modis.gsfc.nasa.gov/data/dataprod/dataproducts.php?MOD_NUMBER=09.

[51] Loupian E., Savin I., Bartalev S., Tolpin V., Balashov I., Plotnikov D. (2011). Satellite service for vegetation monitoring VEGA. Cur. Prob. Remote Sens. Earth Space.

[52] Bartalev S.A., Isaev A.S., Lupyan E.A. (2008). Priorities of satellite monitoring of boreal ecosystems. Contemp. Probl. Ecology.

[53] Bartalev S.A., Belward A.S., Erchov D.V., Isaev A.S. (2003). A new SPOT4-VEGETATION derived land cover map of Northern Eurasia. Int. J. Remote Sens..

[54] SPSS Software. http://www-01.ibm.com/software/analytics/spss.

[55] IBM SPSS Decision Trees 19. http://www.csun.edu/sites/default/files/decision-trees19.pdf.

[56] Zehender G., Ebranati E., Bernini F., Lo Presti A., Rezza G., Delogu M., Galli M., Ciccozzi M. (2011). logeography and epidemiological history of West Nile virus genotype 1a in Europe and the Mediterranean basin. Infect. Genet. Evol..

[57] Lvov D.K., Butenko A.M., Gromashevsky V.L., Kovtunov A.I., Prilipov A.G., Kinney R., Aristova V.A., Dzharkenov A.F., Samokhvalov E.I., Savage H.M. (2004). West Nile virus and other zoonotic viruses in Russia: Examples of emerging—Reemerging situations. Arch. Virol..

[58] Uejio C.K., Kemp A., Comrie A.C. (2012). Climatic controls on West Nile virus and Sindbis virus transmission and outbreaks in South Africa. Vector Borne Zoonotic Dis..

